# Nonlinear response of Q-boosting metasurfaces beyond the time-bandwidth limit

**DOI:** 10.1515/nanoph-2022-0082

**Published:** 2022-05-18

**Authors:** Pavel A. Shafirin, Varvara V. Zubyuk, Andrey A. Fedyanin, Maxim R. Shcherbakov

**Affiliations:** Faculty of Physics, Lomonosov Moscow State University, Moscow 119991, Russia; Department of Electrical Engineering and Computer Science, University of CA, Irvine 92697, CA, USA

**Keywords:** metasurfaces, nonlinear optics, semiconductors, time-varying systems

## Abstract

Resonant nanostructures, such as photonic metasurfaces, have created an unprecedented case for enhanced light–matter interactions through local field engineering. However, the presence of resonances fundamentally limits the bandwidth of such interactions. Here, we report on amending the nonlinear optical response of a semiconducting metasurface through Q-boosting, where the Q-factor of a metasurface rapidly increases with time. The coupled-mode theory reveals overcoming the bandwidth limit by coupling a broadband signal to a bandwidth-matched resonance and Q-boosting at a timescale faster than a resonator lifetime. A control–excitation experiment simulation using a tailored Q-boosting silicon-germanium metasurface predicts the third-harmonic enhancement by factors of 8 (peak) and 4.5 (integrated) against the best-case static metasurface. An analysis of free-carrier losses based on experimental data shows robustness to nonradiative losses and offers a viable pathway to increasing the light–matter interactions beyond the bandwidth limit, with implications in nonlinear and quantum optics, sensing, and telecommunication technologies.

## 1 Introduction

Despite their subwavelength dimensions, resonant nanophotonic structures have provided a groundbreaking route to enhanced light–matter interactions through high-quality-factor (high-Q) resonances, such as Fano resonances [1, 2] and bound states in the continuum [3–5]. High-Q structures have proven to be successful in many instances, such as in enhancement of nonlinear processes [6–16], light emission [17, 18], quantum light generation and manipulation [19], coupling to quantum emitters [20], topological photonics [21], and optical sensing [22, 23]. However, in most resonators, light trapping only occurs within a narrow bandwidth of ≈ω/Q , where *ω* is the natural frequency of the mode and *Q* is its Q-factor. This limitation signifies the so-called time-bandwidth (or delay-bandwidth) limit for linear time-variant systems [24]. It impedes nanophotonic interactions with ultrashort laser pulses, thermal radiation, luminescence, and other broadband light sources.

The time-bandwidth tradeoff can be overcome in time-variant photonic systems. In the recent surge of interest [25], time-variant resonators have extended the scope of resonant light–matter interactions for the purposes of coupling beyond the time-bandwidth limit [26–29], frequency conversion [30–36] and spectral bandwidth manipulation [37], pulse generation and compression [38, 39], and time reversal [40]. In particular, the idea of using a resonator with a growing Q-factor was proven, through coupled-mode theory calculations, to enable indefinite energy storage [28] and capturing broadband light into high-Q modes [29]. Experimentally, such temporal modifications can be enabled through ultrafast optical pumping [41–46]. Optically induced reduction of the Q-factor has been demonstrated to induce frequency conversion [47] and highly tunable THz response [48]. However, metasurfaces with dynamically growing, optically controlled Q-factor require delicate balancing between radiative and nonradiative losses, and, therefore, have not been demonstrated yet.

Here, we devise a semiconductor photonic metasurface with a dynamically increasing quality factor—a Q-boosting metasurface. The Q-boost is accomplished via the photoinduced modulation of radiative losses in the resonator, enabled by an imbalanced two-nanoparticle unit cell. Using a composite silicon-germanium design, we induce inhomogeneous photoinduced modulation of the refractive index in the germanium part of the sample, restoring the radiative balance between the unit cell constituents. This results in a numerically verified Q-boost by a factor of *Q*_+∞_/*Q*_−∞_ ≈ 3, where *Q*_+∞_ and *Q*_−∞_ are the final and initial Q-factors, respectively. A coupled-mode theory (CMT) with time-variant radiative losses shows multifold enhancement of the energy coupled to the resonator when a broadband pulse arrives around *τ* ≈ *τ*_−∞_ = *Q*_−∞_/*ω* before the Q-boost, where *ω* is the natural frequency of the resonator and *τ*_−∞_ is the initial lifetime of the mode. After the Q-boost, the interaction between the metasurface and the broadband light pulse occurs beyond the time-bandwidth limit. We employ these results to demonstrate third-harmonic generation (THG) enhancement of up to a factor of 11 against the best-case scenario attainable with a time-invariant resonant metasurface. We verify the CMT results by finite-difference time-domain simulations with explicitly time-variant materials implemented. The results are consistent with the CMT, showing up to 8 (peak) and 4.5 (integrated) THG enhancement for an experimentally realistic Q-boost from *Q*_−∞_ = 160 to *Q*_+∞_ = 480. Based on the available experimental data, we have also carefully analyzed the free-carrier induced nonradiative losses in the constituent semiconductors and concluded they are small enough to enable an experimentally achievable Q-boost. Introducing resonances dampened by free-carrier losses in germanium reduces the Q-boost to *Q*_+∞_/*Q*_−∞_ ≈ 2 and the THG enhancement to ≈1.9 . Further steps to optimizing the performance of realistic Q-boosting metasurfaces are discussed. Q-boosting metasurfaces take the next step toward a compact platform for light–matter interactions beyond the time-bandwidth limit and with the potential to become an enabling technology in nonlinear and quantum optics, sensing, and telecommunications.

## 2 Results

### 2.1 Idea

**Figure 1:**
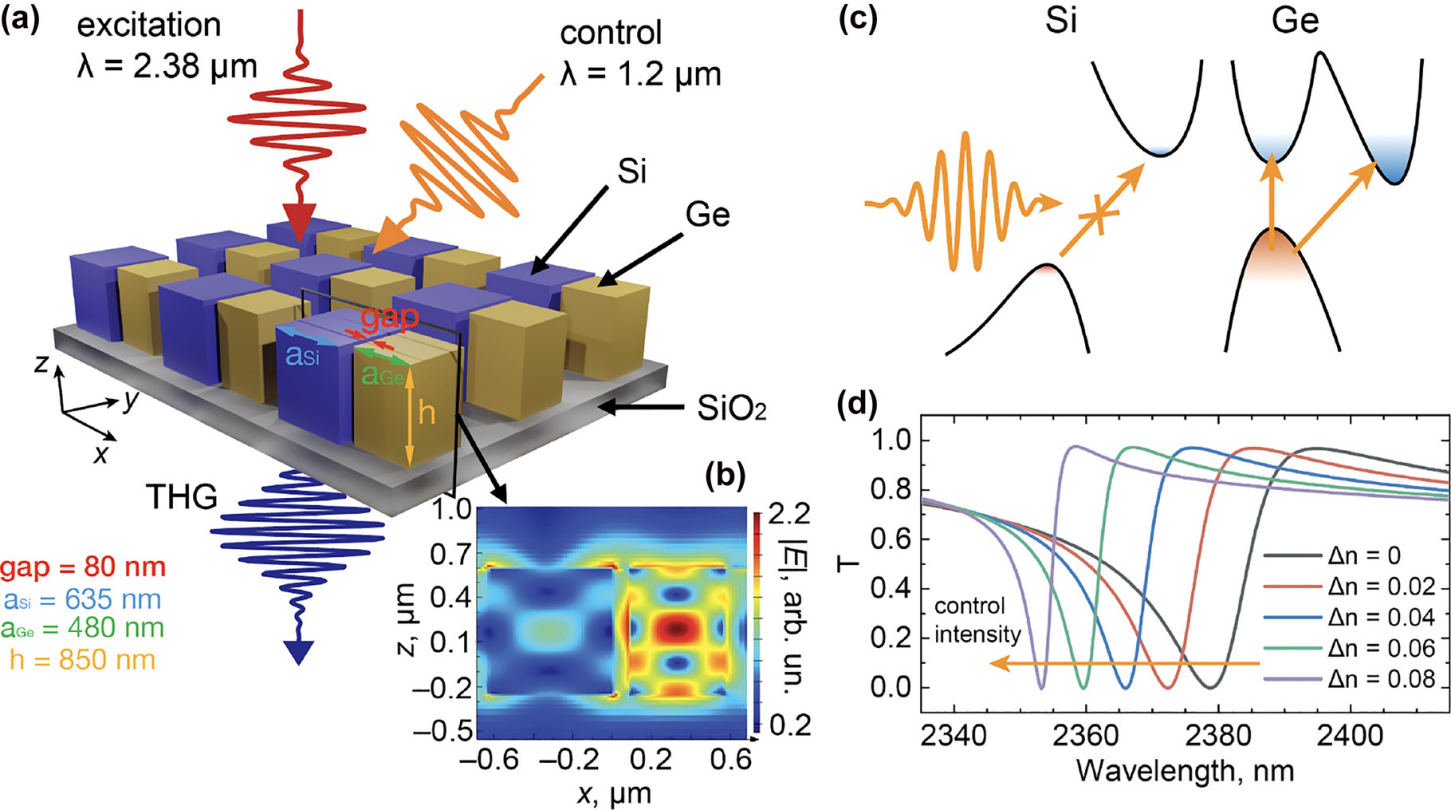
Q-boosting metasurface design. (a) A schematic image of a Q-boosting metasurface. The unit cell consists of germanium and silicon pillars of square cross-section with a side of *a*_Si_ = 635 nm for silicon and *a*_Ge_ = 480 nm for germanium. The height of the structure is *h* = 850 nm. The gap between the pillars is 80 nm. (b) The electric field of a quasiguided mode inside a unit cell of the metasurface at a resonant wavelength of 2380 nm. (c) Schematically, the control pulse produces photogenerated free carriers in the Ge nanoparticles while the Si nanoparticles remain unchanged, as the control photon energy is smaller than the bandgap of Si. The reduction of the Ge refractive index balances the dipole moments of the pillars, boosting the mode Q-factor. (d) Transmission spectra of the metasurface for various Δ*n* in germanium. The narrower resonant curves for lower refractive index values demonstrate a controlled Q-factor boost.

The idea of Q-boosting metasurfaces roots in the fact that a broadband laser pulse can efficiently interact with a narrowband resonator beyond the time-bandwidth limit. This is done through coupling to a low Q-factor mode with the Q-factor growing while the light is trapped in the resonator, thereby prolonging the interaction time without sacrificing the bandwidth. The proposed idea of a Q-boosting semiconductor metasurface is shown in Figure 1a. The unit cell design includes two disparately sized semiconductor pillars made of two different semiconductor materials, e.g., Si and Ge. Full-wave simulations with a commercial solver (Ansys Lumerical FDTD) reveal a quasiguided mode [49] at a wavelength of 2380 nm with a Q-factor of 160, manifested as a dip in the transmission spectrum, shown with the black curve in Figure 1d. The excited mode, illustrated with the local E-field for the resonant wavelength in Figure 1b, carries uncompensated dipole moments in the two cuboids. Therefore, the Q-factor of the resonance depends on the refractive index contrast between the two materials. To dynamically control the asymmetry between the pillars, two materials with different bandgaps are chosen, allowing one to excite free carriers in one of the pillars while leaving the other unchanged (Figure 1c). The refractive index of germanium is controlled through free carrier generation, which can be done through optical pumping within a photon energy window of roughly 0.66–1.12 eV, above the bandgap of Ge and below the bandgap of Si. For example, at a control wavelength of 1.2 μm, the absorption constant of Ge *α*_Ge_ = 1.1 × 10^4^ cm^−1^, and for a carrier density of *N* = 5 × 10^19^ cm^−3^ one would need a typical fluence of about $F=NhE/2\left(1-{\text{e}}^{-\alpha_{\text{Ge}}h}\right)\approx 1$ mJ/cm^2^ [41]; here, *h* is the metasurface’s thickness, and *E* is the control photon energy.

With appropriate metasurface dimensions, reducing the refractive index will lower the effective light path in the germanium and make the mode more symmetric, lowering the radiative losses and boosting the Q-factor. This effect can be seen in Figure 1d, where transmission curves of the metasurface are plotted for different changes to the refractive index of germanium. Along with a blue shift of the resonance, we see a significant Q-factor boost for realistic values of Δ*n*_Ge_ of up to −0.08 experimentally demonstrated earlier [50, 51]. In what follows, we present several categories of cases studied in this paper: the lossless CMT cases, reporting a wide variety of Q-boost amplitudes, aiming at giving a broad perspective on the achievable nonlinear enhancements; a lossless full-wave simulation, with a specific geometry of a metasurface providing Q-boosts of up to 3 to gauge typical refractive index modulations needed to reach such a Q-boost; and lossy CMT cases, informed by lossy full-wave calculations, where a maximum Q-boost of 2.3 is achieved for an experimentally realistic set of material parameters.

### 2.2 Coupled-mode theory: lossless case

Here, we first study a time-variant metasurface schematically depicted in Figure 1a using a lossless CMT with a time-variant Q-factor. At this stage, we make two important approximations. First, the nonradiative losses are neglected; the role of realistic free-carrier-induced losses will be discussed in Section 2.5. Second, the shift in the position of the metasurface’s resonance is also neglected. The benefits of using the dynamic position of the resonance for the purposes of overcoming the time-bandwidth limit in metasurfaces have been discussed in our previous work [52] and its inclusion is beyond the scope of this paper. Using the information on the available refractive index changes, we employ a time-variant CMT, where the conventional CMT [53] is amended by explicitly time-variant parameters [28, 52, 54]:

(1) $$\dot{a}\left(t\right)+\left[i\omega +\gamma_{\text{nr}}\left(t\right)+\gamma_{r}\left(t\right)\right]a\left(t\right)=\sqrt{\gamma_{r}\left(t\right)}s^{+}\left(t\right).$$

Here, *a*(*t*) is the complex amplitude of the excited mode, *ω* is the resonant frequency of the mode, *s* ^+^(*t*) is the excitation pulse and *γ*_r_(*t*) and *γ*_nr_(*t*) are the time-dependent radiative and nonradiative contributions to the decay rate. The excitation is taken to be a Gaussian pulse: $s^{+}\left(t\right)=A\left(t\right){\text{e}}^{-\text{i}\omega_{p}t}$ , where the amplitude is given by $A\left(t\right)=A_{0}\exp\left[-{\left(\frac{t-\tau }{\sigma_{p}}\right)}^{2}\right]$ , *ω*_p_ is the pulse central frequency, and *σ*_p_ is its duration. Time *τ* denotes the pulse arrival time. In practice, for *γ*_r_(*t*) and *γ*_nr_(*t*), we envision the temporal switching enabled by absorbing of another short laser pulse that creates photogenerated free carriers. Hence, for a Gaussian control, assuming a linear dependence of free carrier concentration *N*(*t*) on *γ*_nr_(*t*) and *γ*_r_(*t*), the dynamics can be modeled by an error function centered around *t* = 0: $\gamma_{r}\left(t\right)=\gamma_{\text{r0}}+\frac{\Delta \gamma_{r}}{2}\left(erf\left(t/\sigma \right)+1\right)$ , where *γ*_r0_ is the starting value of the radiative decay rate, Δ*γ*_r_ determines how much the decay rate changes by and *σ* controls how fast the change happens. We assume the dependence for *γ*_nr_(*t*) will have a similar form with the exception that *γ*_nr_(−∞) = 0, since absorption can be neglected in semiconductors for photon energies below the band gap.

**Figure 2:**
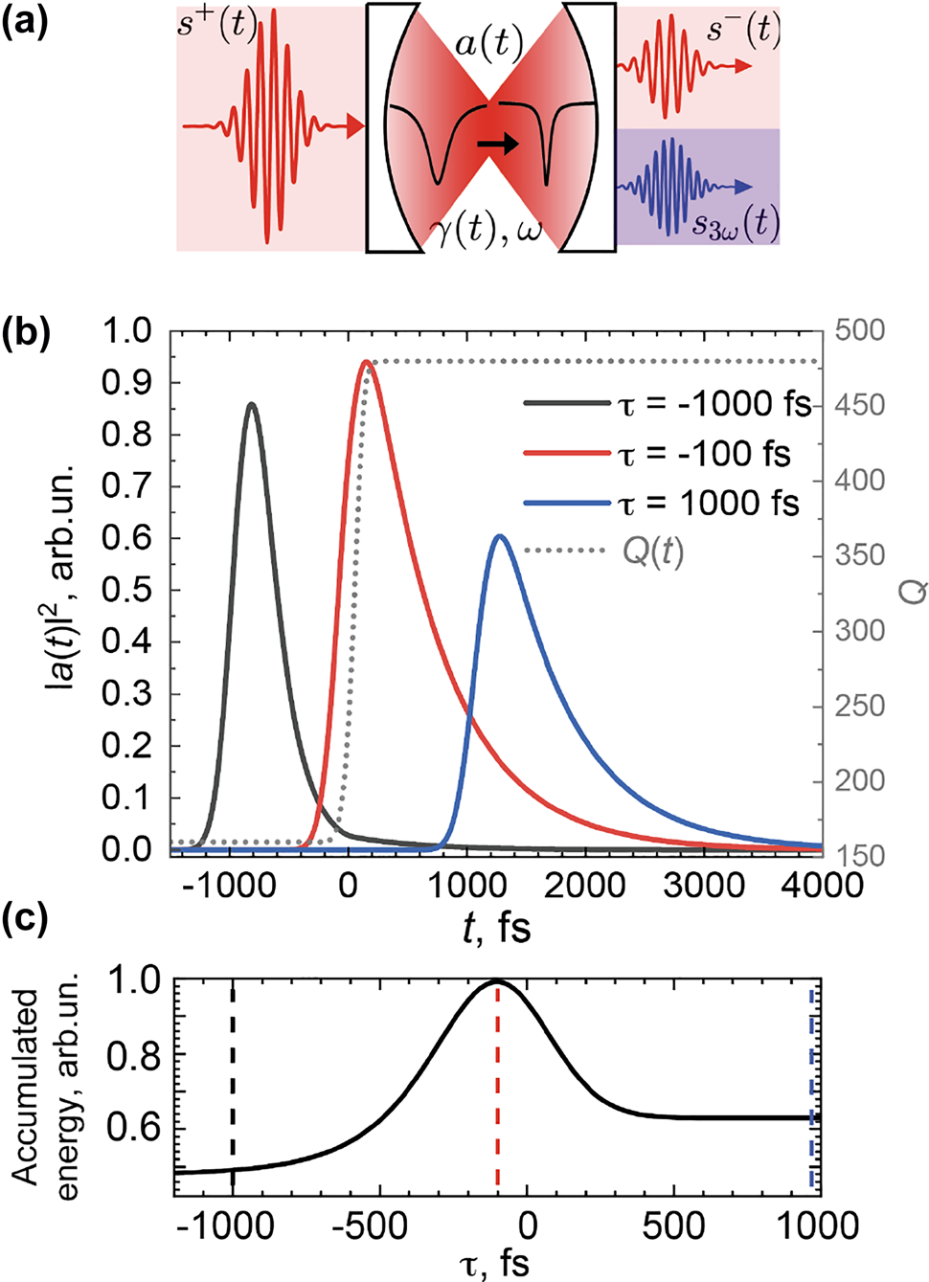
CMT results. (a) Formulation of the coupled-mode theory for a nonlinear Q-boosting metasurface. (b) Mode amplitude for three different values of the control–excitation delay *τ*: −1000 fs, where the resonance is excited before the Q-boost; −100 fs, where the Q-factor boosts right after the resonance is excited; and 1000 fs, where the excitation pulse arrives after the Q-boost. The dotted grey curve shows the time-dependent Q-factor. (c) Energy within the resonator $\left(U\left(\tau \right)=\int_{-\infty }^{+\infty }|a\left(t,\tau \right){|}^{2}dt\right)$ as a function of *τ*, indicating the advantage of Q-boosting at *τ* ≈ −100 fs. The dashed lines indicate the cases from panel (b) in corresponding colors (black, red, blue).

For the sake of example, we solve Eq. (1) with the following parameters: *ω* = *ω*_p_ = 791 ps^−1^ (*λ* = 2.38 μm), *σ*_p_ = 240 fs (pulse full-width at half-maximum *σ*_FWHM_ = 400 fs), *γ*_r0_ = 2.5 ps^−1^, *γ*_nr0_(*t*) = 0 ps^−1^, Δ*γ*_r_ = −1.67 ps^−1^, *σ* = 50 fs. The choice of parameters is justified by existing control–excitation techniques with excitation pulses in the near- to mid-infrared and the necessity to work well below the band gap for both semiconducting materials. With these parameters, the resonance has a starting Q-factor of *Q*_−∞_ = 160, and becomes 3 times larger after the control pulse arrives near *t* = 0: *Q*_+∞_ = 480. The excitation pulse has a spectral width of 20 nm, slightly larger than the initial resonance bandwidth of 15 nm, and significantly larger than the final bandwidth of 5 nm. In Figure 2b, we plot |*a*(*t*)|^2^ for three different values of *τ*. The black curve shows the case of the pulse arriving before the Q-boost and therefore interacting with the unchanged resonance. Here, the pulse effectively couples to the resonance of a similar bandwidth but quickly decays due to the short lifetime: by the time of the Q-boost, almost all the energy in the mode escapes through radiative losses. The blue curve shows the case when the pulse arrives after the Q-boost. The lifetime is long, but the pulse cannot efficiently excite the resonant mode because of the bandwidth mismatch, leading to a lower overall amplitude. The red curve shows the optimal case when the pulse arrives shortly before the Q-boost. Here, the broadband coupling of the pulse to the mode precedes the Q-boost, which happens before the mode has time to decay. This timing traps most of the energy in the high-Q mode and maximizes both the lifetime and overall amplitude of the light in the cavity. Figure 2c plots the overall energy coupled to the cavity over the modeled time period: $U\left(\tau \right)=\int_{-\infty }^{\infty }|a\left(t,\tau \right){|}^{2}dt$ as a function of *τ*. This integral can be used as a measure of interaction between the pulse and the resonator’s material, and we observe that it reaches its maximum at *τ* ≈ −100 fs, signifying the optimal conditions for the Q-boost. The reason *U* peaks near −100 fs is in the scale of the resonator lifetime, which is equal to *Q*_−∞_/*ω* ≈ 200 fs.

### 2.3 Third-harmonic generation

**Figure 3:**
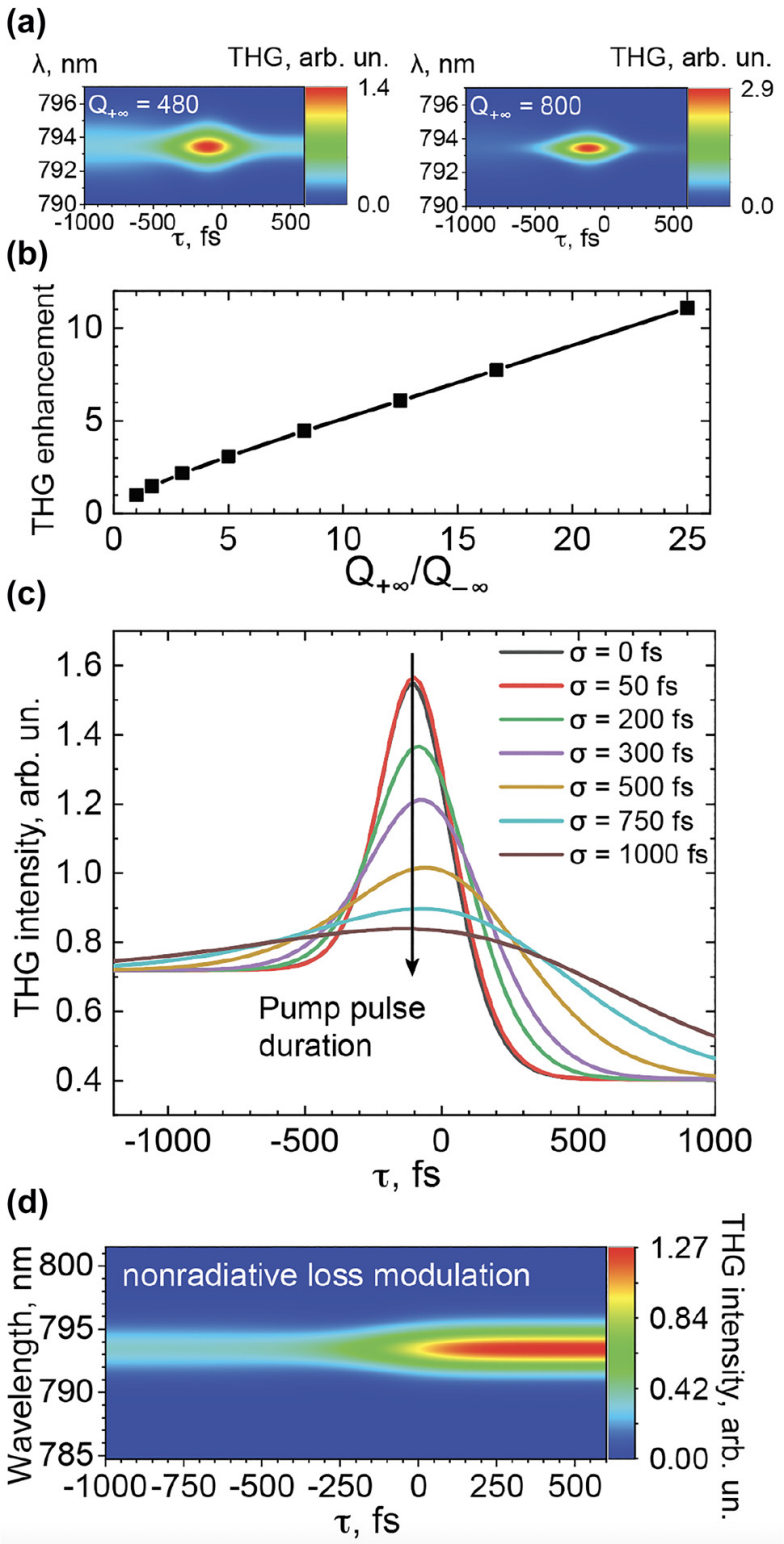
Third harmonic response. (a) Third-harmonic generation (THG) enhancement as a function of the final mode Q-factor (*Q*_+∞_). Insets show THG spectra for *Q*_+∞_ = 480 and *Q*_+∞_ = 800. (b) Integral of the THG spectrum as a function of *τ* for a variety of Q-boost durations *σ*. (c) THG as a function of *τ* with static nonradiative losses and dynamic radiative losses. (d) THG as a function of *τ* with dynamic nonradiative losses and static radiative losses.

As an example of enhanced nonlinear interactions, we will now adopt the Q-boosting approach to the third-harmonic generation (THG) in the metasurface. By modeling the output third-harmonic field as proportional to the third-order polarization, *s*_3*ω*_ = *a* ^3^(*t*), we calculate the THG spectrum through Fourier transform: *I*_TH_ = |FT(*a* ^3^(*t*))|^2^. Although this approach disregards many important factors, such as nonlinear anisotropy and the spatial distribution of nonlinear sources, it serves the purpose of comparing the conversion efficiency in various excitation regimes. The resulting THG spectra are shown in Figure 3a for *Q*_−∞_ = 160 and two values of *Q*_+∞_ = 480 and 800. As expected, we see the strongest harmonic generation at around *τ* = −100 fs, the same delay where the resonant mode shows the maximum overall energy accumulation. The overall spectrally integrated THG enhancement with the Q-boost with respect to a static resonator with *Q*_−∞_ = 160 is 2.1 for *Q*_+∞_ = 480 and 3 for *Q*_+∞_ = 800. A larger boost in the Q-factor creates a greater THG enhancement: Figure 3b shows the THG enhancement as a function of *Q*_+∞_/*Q*_−∞_ varying from 1 to 25 for the optimal value of *τ*. The resulting enhancement is more than an order of magnitude within this range and improves to up to 11 at larger values of *Q*_+∞_. The progress in nanofabrication makes such high Q-factor values a short-term possibility [55].

The Q-boost must happen over a timescale shorter than the initial resonance lifetime. Figure 3c shows the generated THG as a function of *τ* for various values of control duration *σ*. The highest enhancement is achieved as *σ* approaches 0, making *γ*_r_(*t*) a step function. The effect becomes less pronounced as the switching time increases and becomes closer to the original resonance’s lifetime of 200 fs, and on even larger switching times, it gradually vanishes.

The Q-boost must be realized through radiative losses to enhance nonlinearities. The instance of Q-boost realized through nonradiative losses is solved through Eq. (1) with a nonradiative component: *γ*_r0_ = 1.25 ps^−1^, *γ*_nr0_ = 1.25 ps^−1^, Δ*γ*_0_ = 0 ps^−1^, Δ*γ*_nr0_ = −0.8 ps^−1^. Here, *Q*_−∞_ = 160 and *Q*_+∞_ = 235. The resulting THG spectra are shown in Figure 3d. As opposed to the radiative mechanism, we observe merely a gradual change from the original spectrum for *τ* = −∞ to the final state for *τ* = +∞, with no additional enhancement stemming from the dynamic nature of the metasurface.

### 2.4 Full-wave verification

CMT calculations indicate Q-boosting as a viable strategy for enhanced nonlinear interactions between broadband light pulses and resonant metasurfaces. We verify these findings by full-wave simulations introducing to FDTD an explicitly time-variant material. We simulate the response of the metasurface shown in Figure 1a, disregarding material dispersion and assuming the initial germanium and silicon refractive indexes to be 4.1 and 3.4, respectively. Being pumped by an infrared laser pulse, the permittivity of germanium was set to be:

(2) $$\varepsilon \left(t\right)=\varepsilon \left(-\infty \right)-\frac{\Delta \varepsilon }{2}\left[erf\left(\frac{t}{\sigma }\right)\right],$$

where *ɛ*(−∞) = 16.81 is the original permittivity of Ge, Δ*ɛ* = 0.5 is the change in permittivity which leads to a final refractive index of *n*_+∞_ = 4.04 and *σ* = 50 fs is control duration or, analogously, the Q-boost duration. Since we are interested in the THG enhancement, we model the third-order nonlinear response through the cubic nonlinear polarization: *P* = *χE* + *χ* ^(3)^ *E* ^3^. For simplicity, we take the same arbitrary value of *χ* ^(3)^ for both germanium and silicon. In order to satisfy causality, we do not dynamically modulate the charge-induced absorption; the role of free carrier absorption is discussed below. The excitation pulse has the same shape as in the CMT: $s^{+}\left(t\right)=A\left(t\right){\text{e}}^{-\text{i}\omega_{p}t}$ , $A\left(t\right)=A_{0}\exp\left[-{\left(\frac{t-\tau }{\sigma_{p}}\right)}^{2}\right]$ , *ω*_p_ = 783 ps^−1^, *σ*_p_ = 240 fs. These parameters lead to a gradual transition of the resonance from rightmost curve on Figure 1d to the leftmost one, tripling the Q-factor from 160 to 480.

**Figure 4:**
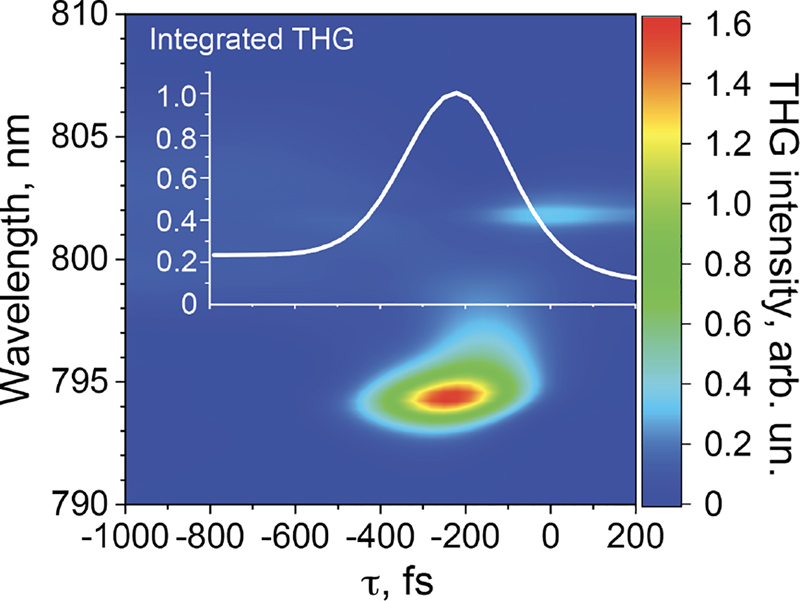
Spectrum of the THG in the metasurface obtained from a dynamic FDTD model plotted versus the delay between the control and excitation *τ*. The inset plot shows the total spectrally integrated THG generated in the metasurface as a function of *τ*.

The resulting THG spectra are plotted on Figure 4 as a function of the control–excitation delay *τ*. We see the generated THG is blue-shifted relative to the original spectrum, as the resonance frequency shift accompanies the Q-boost in our metasurface. This effect is in agreement with our previous studies on overcoming the time-bandwidth limit in time-variant metasurfaces through frequency shift [52]. More importantly, the THG signal is drastically enhanced at a delay of *τ* ≈ 200 fs. The peak spectral intensity value is a factor of ∼8 higher than that of a static, broadband metasurface at *τ* = −1000 fs. The effect of Q-boosting on the overall THG enhancement can be seen in the inset of Figure 4 by integrating the spectra over the wavelength, where we see a 4.5 fold increase in the generated harmonic at *τ* = −250 fs. Note that for the same Q-boost, FDTD gives a greater enhancement value than that in the CMT. This could be explained, in part, by the fact that CMT completely ignores the spatial inhomogeneity of the electromagnetic fields within the resonators’ modes. In full-wave simulations, the formed field hot spots can result in nonlinear emission exceeding that of the homogeneous approximation assumed in the CMT. Therefore, it can be expected that the FDTD-generated THG enhancement is larger than that predicted by the CMT.

### 2.5 Accounting for nonradiative losses

**Figure 5:**
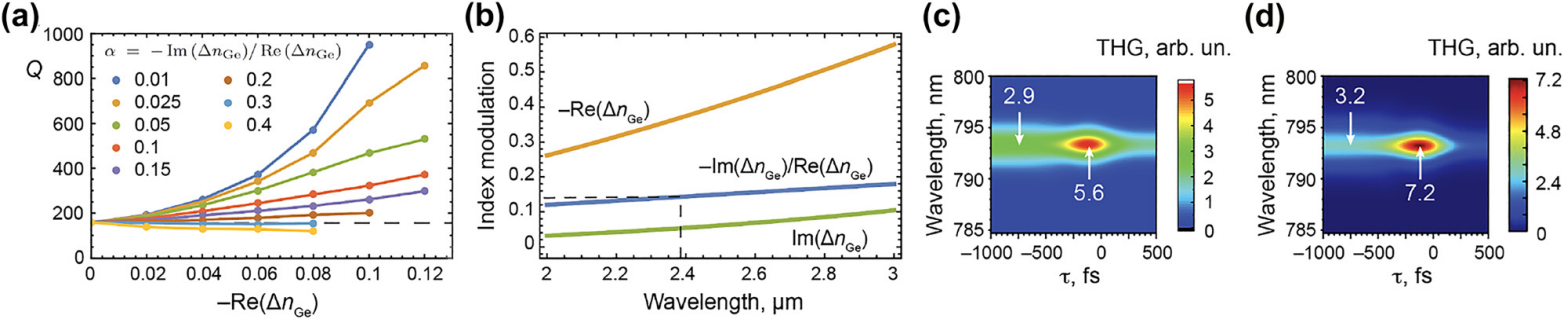
Q-boost in a lossy metasurface. (a) Q-factor of a metasurface with free-carrier losses as a function of Re(Δ*n*_Ge_) and Im(Δ*n*_Ge_). For *α* = −Im(Δ*n*_Ge_)/Re(Δ*n*_Ge_) < 0.3, the overall change of the resonance Q-factor is net-positive. (b) Reference data on Re(Δ*n*_Ge_) and Im(Δ*n*_Ge_) from experimentally acquired plasma frequency of *ω*_pl_ = 1.32 × 10^14^ s^−1^ and free carrier damping frequency Γ = 9.4 × 10^13^ s^−1^ [51]. *α* = 0.14 at the wavelength of 2.38 μm. (c) THG spectrum as a function of the control–excitation delay for Re(Δ*n*_Ge_) = 0.12 and the loss parameter *α* = 0.15; the THG enhancement is 5.6/2.9 ≈ 1.9. Here, the Q-boost is *Q*_+∞_/*Q*_−∞_ = 1.9. (d) Same for the Q-boost of *Q*_+∞_/*Q*_−∞_ = 3; the THG enhancement is 7.2/3.2 ≈ 2.3.

It is known that photogenerated free carriers significantly contribute to both the real and imaginary parts of the refractive index of the semiconductor. In the mid-infrared, far from the semiconductor bandgap, this is primarily done through the Drude dispersion term:

(3) $$\varepsilon \left(\omega \right)=\varepsilon -\frac{\omega_{\text{pl}}^{2}}{\omega^{2}+i\Gamma \omega },$$

where *ɛ* is the high-frequency permittivity, $\omega_{\text{pl}}=\sqrt{Ne^{2}/\varepsilon_{0}m^{*}}$ is the plasma frequency, *N* is the free carrier concentration, *e* is the electron charge, *ɛ*_0_ is the permittivity of a vacuum, m* is the effective carrier mass, and Γ is the free carrier scattering rate which is the primary source of nonradiative losses in the photoexcited semiconductor. Therefore, it is important to verify the robustness of Q-boosting to the free carrier injection with the associated losses. Figure 5a shows the full-wave calculation results of the resonance’s Q-factor as a function of the real modulation of the refractive index Re(Δ*n*_Ge_) and its ratio to the imaginary part Im(Δ*n*_Ge_). For all Re(Δ*n*_Ge_) values, *α* = 0.3 signifies a threshold value beyond which no Q-boost is observed. Note that this threshold is specific to the metasurface design and can be improved by changing the geometry in Figure 1a. Figure 5b plots the real and imaginary index modulations calculated with Eq. (3) for the experimentally attainable values of *ω*_pl_ = 1.32 × 10^14^ s^−1^ and Γ = 9.4 × 10^13^ s^−1^ [51]. The ratio *α* = 0.14 at the wavelength of 2.38 μm is comfortably low to enable efficient Q-boosting, as data for *α* = 0.1 and *α* = 0.15 in Figure 5a indicates.

To verify the degree of applicability of our CMT calculations to lossy Si–Ge metasurfaces, the calculated values of losses and loss-induced decrease of the Q-factor were applied to Eq. (1). As an example, Figure 5c shows the results of calculations with −Re(Δ*n*_Ge_) = 0.12. Here, the loss coefficient was set to *α* = 0.15, and the observable Q-boost is *Q*_+∞_/*Q*_−∞_ ≈ 1.9 (see panel (a)). The calculation shows net-positive THG enhancement near the zero delays, with the enhancement factor being 1.9. Figure 5d shows the same calculation made for a Q-boost of *Q*_+∞_/*Q*_−∞_ ≈ 3, with the proportional nonradiative losses included in the CMT. Here, the peak THG enhancement has increased to up to 2.3. As expected, these figures are smaller than those obtained in the lossless case. Nevertheless, these results show a significant degree of robustness of Q-boosting against nonradiative free carrier losses. Using other semiconductor materials with lower nonradiative free-carrier losses will have a significant impact on the overall performance of nonlinear Q-boosting metasurfaces.

## 3 Discussion

The results above indicate that Q-boosting is a promising mechanism for light–matter interactions beyond the time-bandwidth limit. Even for lossy metasurfaces, a considerable THG enhancement can be observed. Additionally, there are additional steps that can be taken further to optimize the enhancement figures. As an example, the materials of the metasurface can incorporate high-mobility semiconductors instead of Ge. Using GaAs as a host for free carriers can improve the scattering rate from tens of femtoseconds to hundreds of femtoseconds [56], reducing the nonradiative losses by a similar amount. The material engineering aspect of the metasurfaces design will play a crucial role in optimizing their nonlinear optoelectronic response.

Our analysis indicates the experimental feasibility of the Q-boosting scheme proposed in this work and solidifies the Q-boosting technique to significantly enhance the nonlinear optical response of metasurfaces. Finally, as opposed to our previously proposed scheme [52], the present technique does not require chirped signals and therefore has a much wider application scope for low-coherence light sources such as thermal radiation or fluorescence. Q-boosting metasurfaces can be applied to other types of photonic resonators and nonlinearities, including but not limited to high harmonic generation, nondegenerate wave-mixing, self-effects, frequency comb generation, as well as quantum photonics emerging through parametric nonlinearities.

## 4 Conclusions

To conclude, we have proposed a Q-boosting semiconductor photonic metasurface, where the resonance Q-factor sharply increases as a function of time. The Q-boost is accomplished via the photoinduced modulation of radiative losses in the resonator, enabled by a composite dual-semiconductor design. A coupled-mode theory with time-variant radiative losses shows multifold enhancement of the energy coupled to the resonator when a broadband pulse arrives around *τ* ≈ −*τ*_−∞_, the initial lifetime of the mode. After the Q-boost, the interaction between the metasurface and the broadband light pulse occurs beyond the time-bandwidth limit. In the lossless case, we employ these results to demonstrate a third-harmonic generation enhancement of up to a factor of 11 against the best-case scenario attainable with a time-invariant resonant metasurface. Finite-difference time-domain simulations with explicitly time-variant material properties have verified our strategy, showing up to 8 (peak) and 4.5 (integrated) THG enhancement for an experimentally realistic Q-boost from *Q*_−∞_ = 160 to *Q*_+∞_ = 480, where *Q*_+∞_ and *Q*_−∞_ are the final and initial Q-factors of the resonance, respectively. Based on the available experimental data, we have also carefully analyzed the free-carrier induced nonradiative losses in the constituent semiconductors. By introducing commensurate nonradiative losses to the coupled-mode theory, we found decreased Q-boosts and harmonic enhancements of 2 and 1.9, respectively. Possible routes for loss mitigation and Q-boost optimization have been discussed. Q-boosting metasurfaces take the next step toward a compact platform for light–matter interactions beyond the time-bandwidth limit and with the potential to become an enabling technology in nonlinear and quantum optics, sensing, and telecommunications.

## Supplementary Material

Supplementary Material Details
