# Supplementary material for: Nonlinear response of Q-boosting metasurfaces beyond the time-bandwidth limit
Source: Nanophotonics. 2022 May 18;11(17):4053–61. doi: 10.1515/nanoph-2022-0082 (PMC11501905; doi:10.1515/nanoph-2022-0082)
Supplement: Supplementary file 1 — Supplementary Material Details [file j_nanoph-2022-0082_suppl.pdf]

Supplementary information

Nonlinear response of Q-boosting metasurfaces beyond the time-bandwidth limit

Pavel A. Shafirin,[1] Varvara V. Zubyuk,[1] Andrey A. Fedyanin,[1] and Maxim R. Shcherbakov[2]

[1] *Faculty of Physics, Lomonosov Moscow State University, Moscow 119991, Russia*
[2] *Department of Electrical Engineering and Computer Science, University of California, Irvine, CA 92697, USA*

Nanofabrication flows

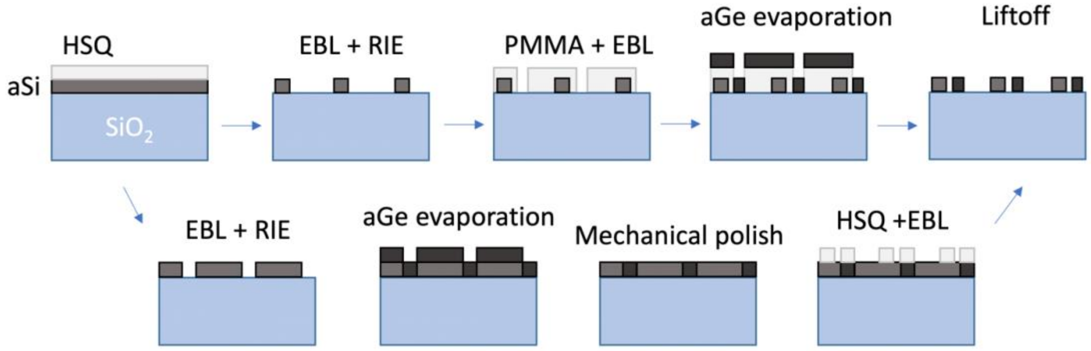

Figure S1. Two potential nanofabrication flows for dual-semiconductor Q-boosting metasurfaces.

Here, we outline two potential nanofabrication flows that could be used to produce the dual-semiconductor Q-boosting metasurface.

The first process starts with defining the final silicon pillars in an amorphous film by using electron beam lithography (EBL) with a negative resist such as HSQ and subsequent reactive ion etching (RIE). Almost ideally vertical sidewalls in this fabrication process can be obtained by using Cl- or Br-based plasmas. In the following step, a layer of positive resist, such as PMMA, is deposited. By using alignment marks, the exposure is done where the germanium pillars are supposed to be placed. After development, a thermally evaporated layer of amorphous germanium is deposited. The final structure is obtained by the final liftoff step where the PMMA and germanium above it are removed in a solvent. The advantage of this approach is in its simplicity, while the disadvantage is in the final non-vertical sidewalls of thermally evaporated germanium pillars.

The second process starts with defining hole-type structures in the silicon layer where the germanium pillars are supposed to be located. After the RIE, germanium is thermally evaporated over the holey silicon film. The top layer of germanium is then removed by mechanical polish. Finally, the silicon pillars are formed using an additional step of EBL, where the future germanium and silicon pillars are masked with a negative resist. The advantage of this method is in potentially near-perfect sidewalls, while the disadvantage is in the difficulty of precise mechanical removal of the top germanium layer in step 3.
